# Effects of orange peel powder on rheological properties of wheat dough and bread aging

**DOI:** 10.1002/fsn3.2080

**Published:** 2020-12-25

**Authors:** Lihong Han, Jiajia Zhang, Xiaohong Cao

**Affiliations:** ^1^ Collaborative Innovation Center for Food Production and Safety School of Biological Science and Engineering North Minzu University Yinchuan China; ^2^ Ningxia Ruichun Coarse Cereals Co., Ltd. Guyuan China

**Keywords:** bread aging, orange peel powder, rheo‐fermentation, thermomechanical characteristics, viscoelastic, wheat dough

## Abstract

Orange peels, the major byproduct of orange fruit processing, are a good material for functional food production because of their excellent physiological and health function. The effects of orange peel powder (OPP) on the rheological and reho‐fermentation properties of high‐gluten wheat dough and bread staling were investigated. The results showed that OPP significantly modified wheat dough characteristics and bread quality for its fiber, pectin and polyphenol content. Incorporation of OPP in wheat dough mainly caused competitive water absorption. It improved dough water absorption from 59.70% to 66.82% by increasing the development time (from 1.40 min to 4.51 min) and decreasing the retrogradation degree (from 1.01% to 0.68%) at a low content (no more than 5%) but showed adverse effects at higher content because of stronger gluten‐dilution action than excessive water sequestration of OPP. It strengthened the dough elasticity by increasing the value of storage modulus (G′) and loss modulus (G″) of dough samples at all contents, G′ and G″ value of dough sample containing 7% OPP was more than twice that of the wheat dough. Alveograph and rheofermentographic parameters confirmed that OPP improved the total volume of CO_2_ production from 1774.11 ml (wheat dough) to 2,458.30 ml (dough sample containing 7% OPP) but reduced the gas retention coefficient from 71.86% to 66.52% during fermentation accordingly. Additionally, no remarkable deterioration of the bread staling was observed. These results contributed to the interpretation of the action mechanism of OPP modification on the wheat dough structure and further guided the application of OPP on cereal product development.

## Graphical abstract

OPP significantly modified wheat dough characteristics and bread quality because of its fiber, pectin, and polyphenol content. Substitution of OPP in wheat dough mainly caused competitive water absorption. It improved dough water absorption by increasing the development time and decreasing the retrogradation degree at a low content (no more than 5%) but showed adverse effects at higher content because of stronger gluten‐dilution action than excessive water sequestration of OPP. It strengthened the dough elasticity by increasing the value of storage modulus (G′) and loss modulus (G″) of dough samples at all contents. Alveograph and rheofermentographic parameters confirmed that OPP improved the total volume of CO_2_ production but reduced the gas retention coefficient during fermentation. Additionally, no remarkable deterioration of the bread staling was observed.

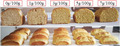

## 1 INTRODUCTION

Functional diets, such as high‐dietary‐fiber diets and polyphenol‐rich diets, have been correlated with a lower risk of many diet‐related diseases, for instance, diabetes, cardiovascular disease, obesity, hypertension, and gastrointestinal disorder. Cereal products, particularly bread, are staple foods that provide opportunities to deliver health benefits to large populations. Therefore, bread is a priority vehicle for enrichment in functional components (Le Bleis et al., 2015). By‐products of fruit have been verified to contain high contents of dietary fiber (O'Shea et al., 2012). As a dough food ingredient, fruit fiber possesses such positive attributes as water holding capacity, high water binding, and good gelling function (Rosell et al., 2009). The addition of by‐products of fruits in bread production has been widely studied, such as apple pomace (Bartkiene et al., 2017), tomato pomace powder (Majzoobi et al., 2011), and carrot pomace (Tańska et al., 2007). Worldwide industrial citrus by‐products have a production quantity of more than 15 million tons, and, during the course of orange fruit processing, the orange peels (*Citrus sinensis* L.) are the major byproduct (Wang et al., 2015). Orange peel contains abundant flavonoids, including C‐or O‐glycosylated flavones, O‐glycosylated flavanones, flavonols, polymethoxylated flavones (PMF,) and other various phenolic acids (Singanusong et al., 2015). Orange flavonoids have various bioactivities, including anti‐inflammatory, antiatherosclerosis, anticarcinogenic, and antioxidant activities (Pan et al., 2010). Additionally, orange peel is a good source of fiber and pectin (Bocco et al., 1998). Orange fiber possesses bioactive functions and is involved in the improvement in intestinal function and health (Chau et al., 2005); it can be used to reduce the levels of residual nitrite and inhibit lipid oxidation in meat products effectively, thereby enhancing their oxidative stability and prolonging their shelf life (Fernández‐Ginés et al., 2003). Therefore, orange peel could be used as potential nutritional fortification sources in the improvement of foods.

The application of orange peel and its sourcing fibers in wheat flour‐based food has been documented in several studies. They are, Jurasová and Kukurová (Kohajdova et al., 2011), reported that orange dietary fiber improved the sensory qualities of biscuits; O’shea et al. (2015) disclosed that orange pomace flour could increase the total dietary intake of a celiac while not lowing the qualities of gluten‐free bread; and Ocen and Xu (2013) revealed that orange by‐products have a great potential for preparation of fiber‐rich breads from frozen wheat dough with a preferable acceptable citrus flavor. Nevertheless, the main types of bread were currently made of fresh wheat dough, and our research team found that orange peel powder (OPP) enhanced DPPH (1,1‐diphenyl‐2‐picrylhydrazyl) radical scavenging ability and improve sensory quality of the fresh‐dough bread significantly (Dai et al., 2018), but there is scarce information referring to its influence on the rheological properties of wheat fresh dough and bread staling. In this context, different portions of OPP by vacuum freeze drying were supplemented to wheat dough, and the effects on the mixolab behavior, alveolab features, rheological characteristics, and fermenting properties of the fresh dough were investigated. Subsequently, the effects on bread staling were assessed by studying the thermal properties of the bread crumb. The results are expected to elucidate the action mechanism of OPP modification on the wheat dough processability and further guide the application of OPP on cereal product development.

## 2 MATERIAL AND METHODS

### 2.1 Materials

High‐gluten wheat flour was kindly provided by Ningxia Saibeixue Flour Mill Co., LTD. Fresh oranges from the growing region of Sichuan (China) were purchased in Xinhua department store. The peel (exocarp and pericarp) of the fresh oranges were cleaned, dried using a vacuum freeze dryer, pulverized by a drug pulverizer, and passed through a 100‐mesh sieve to prepare OPP sample. Active dry yeast was obtained from Angel Yeast Co., LTD.

### 2.2 Compositional analysis of OPP

Moisture content of the OPP was evaluated gravimetrically in an oven at 105°C; ash and total lipids were assessed based on the AOAC method 923.03 and 920.39; protein was determined by the AOAC method 920.87, and the content of protein = total kjeldahl nitrogen × 6.25; total fiber was analyzed by Enzymatic‐Gravimetric procedure using the AOAC Method 985.29, and the content of total dietary fiber = weight (residue) – weight (ash + protein); total carbohydrate was determined by difference (100 – moisture – ash – protein – total lipids). The content of phenolic compounds was analyzed by the Folin–Ciocalteu reagent as the method described by Tavarini et al. (2008). All experiments were performed in triplicate.

### 2.3 Blends of wheat flour and OPP

Wheat flour and a variable proportion of OPP (0, 1%, 3%, 5% and 7%, wheat flour basis, w/w) were fully incorporated by a sample mixer (MR10L; Chopin, Tripette et Renaud, Paris, France). The blends of wheat flour and OPP were then tightly sealed and stored in a dark and dry place.

### 2.4 Thermomechanical properties

The thermomechanical behavior of the flour samples was studied using a Mixolab analyzer (Chopin, Tripette et Renaud, Paris, France) and the “Chopin+” protocol, which evaluated the starch and protein properties of the composite flour during constant temperature mixing and constant heating and cooling. The protocol parameters were as follows: The dough weight was 90 g, and the mixing speed was 80 rpm/min; each sample was heated to 90°C at the rate of 4°C/min after 8 min of mixing at 30°C, cooled to 50°C at 4°C/min after a 7‐min holding period at 90°C, and finally held at 50°C for 5 min. All analyses were performed in triplicate.

### 2.5 Dynamic rheological measurements

The dynamic rheological properties of the dough samples (ratio of the flour sample to water is 100:66, W: W) were determined using a controlled stress rheometer (AR1500ex; TA Instruments). The test system consisted of a 20‐mm parallel plate with a 3‐mm gap. To prevent water evaporation during the process of measurement, the rim of the dough sample was coated with silicone oil. Frequency sweep experiments were conducted at 30°C from 0.01 to 10 Hz to determine the frequency functions of the storage modulus (G´) and loss modulus (Gʺ). Power law equations (Equations (1) and (2)) were fitted to the test data.

(1) $$G^{\prime }=K^{\prime }\omega n^{\prime }$$

(2) G″=K″ωn″

where *G*′ is storge modulus (Pa), *G*″ represent loss modulus (Pa), ω is angular frequency (rad/s), *n*′, *n*″ and *K*′, *K*″ are constants. All rheological analyses were performed in triplicate.

### 2.6 Alveograph experiments

Alveograph tests were conducted by AlveoLab (Chopin Technologies) according to the AACC International Method 54‐30.02. The alveograph parameters automatically reported by the corresponding computer software program were as follows: tenacity or resistance to extension, also called as the pressure inside the dough bubble, as the height of the peak in the alveogram (P, mmH_2_O); dough extensibility as the width of the curve (L, mm); the equilibrium between the tenacity and extensibility that is expressed by their curve configuration ratio (P/L). Dough strength is expressed as the deformation energy (W), which was calculated using Equation (3).

(3) W=1.32×(V/L)×S

where *V* is aeration volume (mm^3^), *L* is the width of the curve (mm), and *S* is the area of the curve (cm^2^). All analyses were replicated on three different days, and the results were indicated as the average value of three replicates.

### 2.7 Rheo‐fermentation experiments

Rheo‐fermentation experiments were performed using a rheofermentometer (Rheo F4, Tripette & Renaud Chopine) according to the AACC Method 89‐01.01. 7 g of instant dried yeast was dispersed in distilled water, and then, the mixture was added to flour (250 g) during dough preparation. The total added water volume depended on the water absorption capacity of the flour samples to produce a dough of optimal consistency. Next, 315 g of prepared dough was placed into a rheofermentometer to determine the total volume (ml), CO_2_ lost volume (ml), retention volume (ml), and retention coefficient R (%) (ratio of the volume that was detained in the dough to the total volume of produced CO_2_) during a 3‐hr experiment.

In order to further verify the results of rheo‐fermentation experiments, the texture of bread was recorded. The making process of bread was as follows: 290 g of wheat or composite flour, 50 ml of pure milk, 130 ml of water, 30 g of sugar, 3.5 g of salt, 20 g of butter, and 3.5 g of instant dried yeast were placed into a bread maker (PE9600; Beijing Zhongxingbaicui Electric Appliance Co. LTD), under the mode of 01 (hefeng/British bread), to prepare bread automatically. All analyses were performed in triplicate.

### 2.8 Thermal characteristic experiments

A differential scanning calorimeter (Netzsch, DSC 214 polyma, Germany) was applied as an oven to simulate the bread baking process in the center of bread crumb according to the method provided by (León et al., 1997). 15–20 mg of dough samples was weighed in aluminum vessels, sealed, heated from 25 to 100°C at 10°C/min, and hold for 5 min at 100°C. After that, samples were stored at 4°C for accelerating the retrogradation of amylopectin during bread staling. After 2, 4, and 7 days of storage, samples were heated in the DSC from 25 to 110°C at 10°C/min, respectively. The parameters recorded were T_o_ (the onset temperature, °C), T_p_ (peak temperature, °C), T_c_ (conclusion temperature, °C), and ΔH (the enthalpy associated to the starch gelatinization or the amylopectin retrogradation, J/g dough product). The retrogradation index (RI) was defined as Equation (4):

(4) $$\text{RI}=\frac{\Delta H_{\text{retrogradation}}}{\Delta H_{\text{gelatinization}}}\times \text{100\textbackslash{}\%}\;$$

All analyses were in carried out in triplicate. An empty sealed aluminum vessel was used as a reference during the process of each experiment.

### 2.9 Statistical methods

Except for the Alveograph and texture test, which were performed using six replicates, all other experiments were repeated three times. ANOVA and Duncan's multiple range tests were performed to determine the significant differences among the dough or bread samples using SPSS software (SPSS Inc.). The figures were plotted by Origin 8.0 (Microcal Software, Northampton, MA).

## 3 RESULTS AND DISCUSSION

### 3.1 Chemical composition

Table 1 illustrates main chemical components of OPP. As expected, the OPP flour was low in protein and total lipids. It was found to be highly fibrous, containing about 40.56% of total fiber. The content of the carbohydrate was 79.23% in OPP. Previous researches reported higher results for total fiber of 64.3% and equal results of 40.47%, but similar results for ash and protein (O’shea et al., 2015). The difference in composition is most attributed to different planting conditions and the fruit maturity. It is worth mentioning that the OPP flour contained a high level of total phenols, 40.32 mg/g.

**TABLE 1:** Chemical composition of OPP^a^

Components	Contents
Moisture (%)	9.05 ± 0.32
Protein (%)	5.88 ± 0.19
Ash (%)	3.89 ± 0.21
Total lipids (%)	1.95 ± 0.14
Carbohydrate (%)	79.23 ± 0.86
The content of total fiber in OPP	
Total fiber (%)	40.56 ± 1.97
The content of total phenols in OPP	
Total phenols (mg/g)	40.32 ± 2.07

^a^ Each value is expressed as the mean of 3 replicates.

### 3.2 Thermomechanical properties

The thermomechanical parameters of wheat dough and wheat dough supplemented with OPP are shown in Table 2. In order to obtain a torque of 1.11 Nm, the water absorption and dough development time (the maximum time) of wheat flour reached 59.70% (14% moisture basis) and 1.49 min, respectively. However, the addition of OPP to wheat flour led to a significant change (*p* < .05) in the water absorption and dough development time. The increase in the amount of OPP, at the successive levels of 1%, 3%, and 5%, caused a remarkable rise in the Mixolab water absorption and dough development time, whereas the amount of 7% OPP led to a significant decrease compared with 5%. Orange peel was proven to be rich in fiber in this study, and the orange peel fiber is made chiefly from pectin (Quiles et al., 2018). Previous studies have shown that the partial replacement of wheat flour by dried fruit by‐products, such as mango peel powder (Ajila et al., 2008), apple pomace powder, and orange pulp powder (Figuerola et al., 2005; Lu et al., 2017), generally resulted in increased water absorption for their high content of pectin, which has a strong ability to bind large amounts of water and form additional hydrogen bonds (Rosell et al., 2001). The dough development time generally increased with the addition of fruit pomace powder for gluten hydration prevention, either by the competition for moisture that comes from the strong water binding capacity of the pectin itself, or by interactions of pectin with gluten (Quiles et al., 2018). The decrease in the water absorption and dough development time of the wheat flour, containing 7% of OPP, may be attributed to the negative effect of gluten dilution and physical disruption covering up the function of pectin.

**TABLE 2:** Effects of OPP on the thermomechanical characteristics of wheat dough^1^

OPP Content (%)	WA (%)	DDT (min)	ST (min)	C_3_ (Nm)	BD (%)	RT (%)
0	59.70 ± 0.26^a^	1.49 ± 0.15^a^	7.53 ± 0.20^c^	1.69 ± 0.02^c^	0.12 ± 0.02^a^	1.01 ± 0.06^c^
1	62.41 ± 0.32^b^	3.17 ± 0.09^b^	7.12 ± 0.16^b^	1.59 ± 0.04^b^	0.14 ± 0.04^ab^	0.87 ± 0.05^b^
3	64.03 ± 0.57^c^	4.66 ± 0.10^c^	6.56 ± 0.37^a^	1.60 ± 0.05^b^	0.19 ± 0.03^b^	0.73 ± 0.07^ab^
5	66.82 ± 0.43^d^	4.51 ± 0.05^c^	6.57 ± 0.22^a^	1.56 ± 0.03^ab^	0.30 ± 0.03^c^	0.68 ± 0.05^a^
7	59.33 ± 0.19^a^	1.40 ± 0.07^a^	6.15 ± 0.26^a^	1.49 ± 0.04^a^	0.32 ± 0.05^c^	1.92 ± 0.04^d^

Abbreviations: BD: Breakdown; DDT: Dough development time; RT: Retrogration; ST: Stability time; WA: Water absorption.

^1^ Each value is expressed as the mean of 3 replicates. Different superscript letters on the results of the same column indicate significant differences (*p* < .05) based on ANOVA and Duncan's multiple range tests.

The stability time represents the dough mixing tolerance, and the dough seemed to become weaker with increasing in OPP addition. This result is consistent with some previous reports (Mildner‐Szkudlarz et al., 2013). It is most likely due to the dilution of the gluten proteins when OPP is added so that the proteins cannot form a consecutive strong network.

C_3_ indicates the maximum viscosity of dough in the heating stage. The value of C_3_ was diminished significantly with the addition of OPP. OPP seems to decrease the swelling powder of starch granules. The reason was likely due to the high water absorption of OPP. As is well known, the starch viscosity was chiefly determined by amylopectin. Due to water absorption by OPP, no adequate moisture was combined with amylopectin molecules, which could not completely form emplastic. Several previous studies have demonstrated that nonstarch polysaccharides could restrict the hydration of amorphous regions of starch granules (Luo et al., 2017).

The breakdown represents the difference between the peak and minimum value of the dough viscosity during heating. The value of breakdown increased significantly (*p* < .05) with the concentration of OPP. It is well known that a lower breakdown viscosity indicates higher stability of the dough, less granule disruption and a higher tendency of starch to resist shear force during heating. It has been also reported that the addition of apple or lemon fiber decreased the resistance and stability of the starch to mechanical stress and heating during the process (Yildiz et al., 2013).

A setback indicates the degree of starch recrystallization, especially the level of amylose molecule rearrangement during the cooling process. The setback value initially decreased and then increased with the increment of OPP addition. When the added content of OPP was no more than 5%, it showed significant inhibition in the retrogradation of wheat dough; however, an opposite tendency was found with a further increase in OPP addition. It was previously indicated that the addition of pectin significantly reduced the setback value of rice starch, and the reduction effect was positively related to the content of glucuronic acid in the pectin (Luo et al., 2017). It was also reported that glucuronic acid is the main monosaccharide of citrus peel pectin (Hosseini et al., 2019). A higher glucuronic acid content of pectin exhibited a longer backbone and the trend of more difficult gyration orientation, which was detrimental for the rearrangement of starch molecules in the limited space in dough. As the addition increases further to 7% levels, OPP showed stronger effects in promoting than in inhibiting the retrogradation behavior. The cause might be mainly attributed to excess OPP forming crystals by intermolecular interaction, which acts as an adhesive between starch polymer molecules to promote retrogradation.

### 3.3 Oscillatory measurements

**FIGURE 1:**
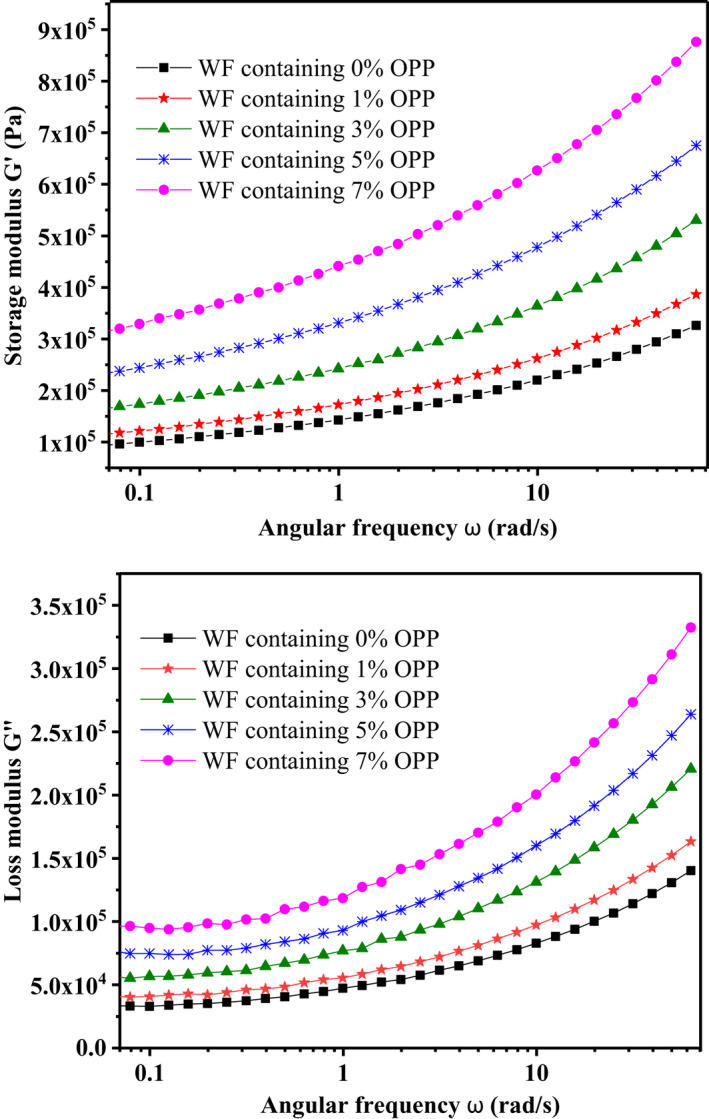
Effect of different proportion of OPP on storage modulus (G′) and loss modulus (G″) of wheat dough (WF, wheat flour)

Information on the viscoelastic properties of the samples is provided by the frequency sweep test. Values of the dough elastic and viscosity are indicated as *G*′ (storage moduli) and *G*″ (loss moduli), respectively, and the *G*′ and *G*″ of the dough samples containing different amounts of OPP are shown as frequencies in Figure 1. In the whole frequency range, *G*″ was lower than *G*′. The *G*′ and *G*″ moduli of all samples increased with the increment in frequency. This rheological behavior indicates that the dough samples are elastic.

Under the same frequency, the value of *G*′ and *G*″ increased drastically as the OPP content increased. These results may be mainly attributed to polyphenols and fiber in OPP. It was well known that the increase in *G*′ was derived from the cross‐linking degree of a polymer system. Proteins can form complexes with polyphenols via hydrogen bonding between a carbonyl group of the peptide residue and a hydroxyl group of phenols (Song & Yoo, 2017), which efficiently fortified the dough networks and improved the viscoelastic moduli in the dough. Additionally, fiber incorporation reduced water lubrication owing to the competition for water absorption between fiber and gluten, and fiber also served as a filler in the dough viscoelastic matrix. Both factors could increase the *G*′ and *G*″ of the dough samples.

### 3.4 Alveograph experiments

**FIGURE 2:**
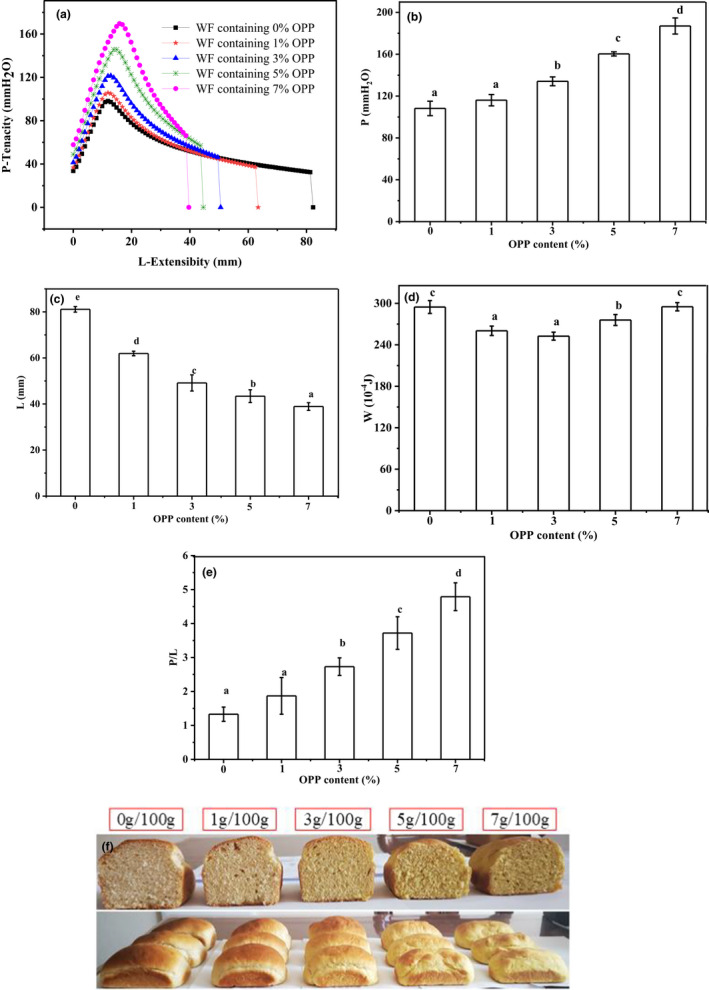
Alveograph curves (a) and the parameters (b, c, d, and e) of wheat dough with different proportion of OPP, and the morphological structures of corresponding bread (f) (WF, wheat flour)

The effect of OPP addition on the alveograph curve and parameters of wheat dough is shown in Figure 2. Addition of OPP resulted in significant changes in wheat dough alveograph properties (Figure 2a), as evidenced by the increase in the resistance to deformation (P, Figure 2b) and gluten performance (P/L, Figure 2c), whereas the reductions (*p* < .05) in extensibility (L, Figure 2e). The index of Alveograph W (Figure 2d) is the value depending on both L and P, so it is not a really reflection of the changes because of OPP addition as are the two single characteristics (Addo et al., 1995). These results are agreeing with the oscillatory measurements. Previous research indicated that the addition of wheat fiber could lead to higher P, P/L, and lower L of wheat dough (Li et al., 2014). Fiber in OPP may have the same effect on wheat dough alveograph characteristics. Moreover, the increment in the P and P/L value was attributed to the increase in gluten strength with an intensification effect of polyphenols in OPP (Song & Yoo, 2017).

### 3.5 Rheo‐fermentation experiments

Dough development and gas retention characteristics were investigated using a rheofermentometer. The rheofermentographic parameters of wheat dough containing OPP are listed in Table 3. The dough maximum height (Hm) in fermentation is directly related to the loaf specific volume of bread; therefore, it is one of the important parameters to evaluate the baking performance of dough samples. The addition of OPP to wheat dough led to a remarkable change in Hm. The amount of OPP, at the level of 1%, caused a rise in Hm, whereas 3% or more of OPP led to a dramatic decrease in Hm compared with that of the control. The increase in Hm may be due to the polyphenols in OPP that enhance the gluten network (Song & Yoo, 2017). Nonetheless, fiber and pectin in OPP, the high water absorption, lead to water migration from the gluten substance and inferior gluten network formation, so that the value of Hm decreased remarkably with the further increase in OPP. The values of (Hm‐H)/Hm were drastically decreased with the increase in OPP, indicating higher dough stability. The cause may be because of the lower Hm of the dough samples with the increase in OPP.

**TABLE 3:** Effects of OPP on the rheofermentographic parameters of wheat dough^1^

OPPC (%)	Hm (mm)	H (mm)	(Hm‐H)/Hm (%)	V_T_ (mL)	V_R_ (mL)	R (%)
0	60.92 ± 0.49^d^	41.8 ± 1.09^d^	31.41 ± 0.99^c^	1774.11 ± 28.15^a^	1,273.46 ± 29.21^a^	71.86 ± 1.22^b^
1	62.35 ± 0.52^e^	46.2 ± 0.69^e^	25.52 ± 1.14^b^	2052.31 ± 34.21^b^	1,337.83 ± 26.88^b^	65.24 ± 0.76^a^
3	44.17 ± 0.41^c^	43.5 ± 0.99^c^	1.67 ± 0.87^a^	1972.56 ± 19.66^b^	1,343.64 ± 17.96^b^	68.18 ± 0.57^a^
5	32.81 ± 0.91^b^	32.7 ± 0.74^b^	2.54 ± 0.96^a^	1944.25 ± 23.08^b^	1,319.34 ± 21.43^b^	67.83 ± 1.78^a^
7	27.93 ± 0.32^a^	26.9 ± 0.87^a^	3.38 ± 1.07^a^	2,458.30 ± 18.25^c^	1634.71 ± 25.15^c^	66.52 ± 0.68^a^

Note

OPPC, the content of OPP; Hm (mm), the maximum height during dough fermentation; H(mm), dough height at the termination; (Hm‐H)/Hm is an index that is negatively related to dough stability; V_T_, total volume of gas produced during fermentation; V_R_, total volume of the gas retained in the dough at the end of fermentation; R (VR/Vt), the gas retention coefficient during fermentation.

^1^ Each value is expressed as the mean of replicates. Different superscript letters on the results of the same column indicate significant differences (*p* < .05) based on ANOVA and Duncan's multiple range tests.

The addition of OPP significantly increased the total CO_2_ production (Vt) of the dough samples compared with that in pure wheat dough. These results were consistent with Coda et al. (2014) and Campbell et al. (2008) who reported that the introduction of wheat fiber into pure wheat dough remarkably increased the total CO_2_ production of the dough. These might be attributed to fiber particles increasing the rate of gas turnover and providing a higher steady‐state void fraction of gas than smaller wheat flour particles, which could promote CO_2_ production in the dough. The gas retention power of dough during fermentation is directly linked to the quality of the gluten network, and it is crucial for bread quality. As shown in Table 3, the CO_2_ volume retained by the dough samples decreased after OPP addition. The decrease of CO_2_ retention resulted in the significant (*p* < .05) decrease of bread volume of the samples containing OPP as the additive amount exceeded 3%. This result can be verified by the bread morphological structure (Figure 2) The increased release of gas may be due to OPP particles disrupting the gluten–starch matrix.

### 3.6 Aging properties

The staling properties of bread containing different proportion of OPP were investigated according to the starch retrogradation of the samples stored at 4°C for 2, 4, and 7 days, respectively, with DSC, and the results were showed in Table 4. Concerning the endothermic characteristics of the gelatinization process of the wheat dough and the dough containing OPP, the addition of OPP promoted a shift trend of the endotherm peak to high temperatures, and a significant (*p* < .05) decrease of enthalpy as the addition of OPP reached to 7% compared to the control sample. Regarding the retrogaradation endotherm, the presence of OPP produced nonsignificant (*p* > .05) influence on endotherm peak compared to the control. As for the retrogradation index, there was nonsignificant (*p* > .05) difference among the control and the samples with OPP stored for 2 or 7 days, respectively, but the values of RI of the samples containing OPP were larger than the control. This result indicated that the samples with OPP have higher aging rate than that of the control during the storage period from 2 to 4 days under the experimental conditions. The mechanism of this results needs further exploration.

**TABLE 4:** Retrogradation properties of baked dough samples stored at 4°C^1^

OPP content (%)	Stored days (day)	T_o_ (^o^C)	T_p_ (^o^C)	T_c_ (^o^C)	△H_r_ (J/g)	RI (%)
0	0	58.33 ± 0.26^e^	66.82 ± 0.89^g^	93.72 ± 0.97^b^	4.30 ± 0.15^d^	0
	2	38.77 ± 1.45^a^	58 ± 0.53de^f^	68.37 ± 0.45^a^	2.89 ± 0.24^ab^	0.67
	4	39.9 ± 0.40^bc^	57.03 ± 0.99^abcde^	68.97 ± 0.93^a^	2.98 ± 0.16^b^	0.69
	7	40.23 ± 0.51^bc^	56.77 ± 0.97^abc^	67.9 ± 0.10^a^	3.18 ± 0.11^b^	0.75
1	0	58.74 ± 0.33^e^	67.80 ± 0.84^gh^	93.77 ± 0.91^b^	4.29 ± 0.37^d^	0
	2	39.60 ± 0.17^b^	57.83 ± 0.65^cdef^	68.03 ± 0.32^a^	2.77 ± 0.07^ab^	0.66
	4	40.23 ± 0.25^bc^	57 ± 0.52^abcd^	68.30 ± 0.66^a^	3.05 ± 0.15^b^	0.71
	7	40.50 ± 0.61^cd^	56.40 ± 0.17^a^	68.00 ± 0.60^a^	3.13 ± 0.09^b^	0.74
3	0	58.99 ± 0.38^e^	68.36 ± 0.68^hi^	93.87 ± 0.45^b^	4.27 ± 0.32^d^	0
	2	40.07 ± 0.38^bc^	58.37 ± 0.32^f^	69.00 ± 0.46^a^	2.90 ± 0.09^ab^	0.68
	4	39.57 ± 1.04^b^	58.17 ± 0.15^ef^	68.97 ± 0.68^a^	3.11 ± 0.27^b^	0.74
	7	39.80 ± 0.10^bc^	56.20 ± 0.20^a^	68.07 ± 0.49^a^	3.16 ± 0.07^b^	0.74
5	0	59.90 ± 0.20^f^	69.33 ± 0.40^ij^	93.97 ± 1.13^b^	4.01 ± 0.11^cd^	0
	2	39.73 ± 0.15^bc^	58.37 ± 0.83^f^	68.77 ± 0.61^a^	2.82 ± 0.17^ab^	0.68
	4	39.9 ± 0.17^bc^	57.03 ± 0.58^abcde^	68.27 ± 0.98^a^	2.93 ± 0.03^ab^	0.74
	7	41.2 ± 0.66^d^	56.57 ± 0.35^ab^	68.40 ± 0.92^a^	3.03 ± 0.37^b^	0.75
7	0	60.15 ± 0.35^f^	69.97 ± 0.46^j^	93.57 ± 0.59^b^	3.90 ± 0.10^c^	0
	2	39.77 ± 0.21^bc^	58.53 ± 0.85^f^	68.77 ± 0.15^a^	2.60 ± 0.08^a^	0.67
	4	40.47 ± 0.57^cd^	57.70 ± 0.40^bcdef^	68.13 ± 0.74^a^	2.94 ± 0.17^ab^	0.74
	7	40.27 ± 0.06^bc^	57.07 ± 0.29^abcde^	67.67 ± 0.23^a^	2.96 ± 0.11^ab^	0.74

^1^ Onset temperature (T_o_), peak temperature (T_p_); conclusion temperature (T_c_); retrogradation enthalpy (*△*H_r_), retrogradation index (RI). All values are indicated as Mean ± Standard deviation. The different superscript letters within each column showed significantly different (*p* < .05) based on ANOVA tests with Duncan's multiple range analysis.

## 4 CONCLUSIONS

The present work demonstrated that OPP significantly modified the wheat dough properties regarding its components of fiber, pectin, and polyphenols. OPP improved the dough water absorption from 59.70% to 66.82% by increasing the development time (from 1.40 min to 4.51 min) and decreasing the retrogradation degree (from 1.01% to 0.68%) at a low content (no more than 5%), but showed adverse effects at 7% content owing to the stronger gluten‐dilution action than the excessive water sequestration of OPP. The viscoelastic profiles indicated that OPP strengthened the dough elasticity by increasing the loss modulus (*G*″) and storage modulus (*G*′) of dough samples at all content levels. Alveograph and rheofermentographic parameters confirmed that OPP improved the total volume of CO_2_ production from 1774.11 ml (wheat dough) to 2,458.30 ml (dough sample containing 7% OPP) but reduced the gas retention coefficient from 71.86% to 66.52% during fermentation accordingly. Meanwhile, OPP had no remarkable influences on the bread staling. This study contributed to interpreting the action mechanism of OPP modification on wheat dough structure and further guided the application of OPP on cereal functional product development.

## CONFLICT OF INTEREST

We declare that we do not have any commercial or associative interest that represents a conflict of interest in connection with the work submitted.

## Data Availability

The data that support the findings of this study are available from the corresponding author upon reasonable request.
